# Developing core competencies for monitoring and evaluation tracks in South Asian MPH programs

**DOI:** 10.1186/s12909-015-0403-5

**Published:** 2015-08-05

**Authors:** Himanshu Negandhi, Preeti Negandhi, Ritika Tiwari, Anjali Sharma, Sanjay Zodpey, Hemali Kulatilaka, Sangeeta Tikyani

**Affiliations:** 1 Indian Institute of Public Health – Delhi, Plot Number 47, Sector 44, Institutional Area, Gurgaon, Haryana 122 002 India; 2 Public Health Foundation of India, Delhi, India; 3 MEASURE Evaluation, University of North Carolina, Chapel Hill, USA

**Keywords:** Public health education, Monitoring and evaluation, Competencies

## Abstract

**Background:**

Monitoring and evaluation (M&E) provides vital information for decision-making and its structures, systems and processes are expected to be integrated throughout the life-cycle of public health programs. The acquisition of these skills should be developed in a structured manner and needs educational systems to identify core competencies in M&E teaching. This article presents our work on harmonizing M&E competencies for Masters level programs in the South Asian context and undertaking the global review of M&E track/ concentration offered in various Masters of Public Health (MPH) programs.

**Methods:**

Through an online search and snow-balling, we mapped institutions offering M&E tracks/ concentrations in Masters of Public Health (MPH) programs globally. We obtained detailed information about their M&E curriculum from university websites and brochures. The data on curricular contents was extracted and compiled. We analyzed the curricular contents using the framework for core competencies developed by the Association of Schools of Public Health (ASPH); and the Miller’s triangle. This data was then used to inform a consultative exercise aimed at identifying core competencies for an M&E track/ concentration in MPH programs in the South Asian context.

**Results:**

Our curricular review of M&E content within MPH programs globally showed that different domains or broad topic areas relating to M&E are covered differently across the programs. The quantitative sciences (Biostatistics and Epidemiology) and Health Policy and Management are covered in much greater depth than the other two domains (Social & Behavioral Sciences and Environmental Health Sciences). The identification of core competencies for an M&E track/ concentration in the South Asian context was undertaken through a consultative group exercise involving representation from 11 institutions across Bangladesh, India, Nepal and Sri Lanka. During the consultation, the group engaged in a focused discussion to reach consensus on a set of 15 core competencies for an M&E track in South Asian MPH programs.

**Conclusion:**

This work presents an opportunity for institutions to identify and re-examine their M&E competencies as a part of their specialized tracks within MPH programs. Our curricular analysis approach has the potential for adaptation and further use in curriculum analysis across different academic specialties.

## Background

Monitoring and Evaluation (M&E) is a powerful management tool that can help both governments and organizations achieve desired results [1]. By providing vital information for decision-making, it assists in reviewing the performance of government policies, programs and projects. While monitoring is the on-going assessment of a project, which measures the progress of a program, evaluation is a periodic measurement of the effectiveness of the project in terms of the objectives it aimed to achieve. M&E has considerable scope in helping organizations to use the results for internal learning and improvement of their work [2].

M&E is vital in the health sector with the World Health Organization (WHO) partnering with several other agencies for development of better M&E systems across countries [3]. Functional M&E systems consist of several components [4] and need people with specialized skills. This is essential because of the complexity of modern public health systems, their need for different types of data from multiple sources spanning across several health system building blocks [5]. M&E structures, systems and processes are expected to be integrated throughout the life-cycle of public health programs [6].

The acquisition of these skills should not just be a natural culmination of ‘experience’ of work within the health system; but needs to be developed with specific and purposive mentoring and training. The presence of trained M&E personnel within health systems is a vital ingredient in building strong, yet flexible M&E systems in the health sector. This M&E capacity is deficient in many developing countries, including India. Postgraduate degree programs such as Masters of Public Health (MPH), which include courses on M&E, aim to offer the training needed to equip M&E leaders with necessary skills. Such programs are being offered in India as well as neighboring countries of Nepal, Sri Lanka and Bangladesh. M&E challenges are ubiquitous across health systems in these four countries.

Education systems represent the supply-side of health systems. From a South Asian perspective, there is widespread recognition that an M&E track or concentration within an MPH program would bring an added value to the program. Our primary objective was to identify the core competencies required for an M&E track/concentration across Masters of Public Health (MPH) programs in South Asia.

## Methods

**Fig. 1:**
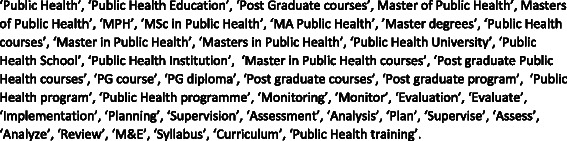
Key words included in the search

We undertook a review of M&E tracks/concentrations available globally to landscape the current teaching in M&E. We also engaged with experts from academics and M&E to evolve South Asia specific M&E competencies through a consultative face-to-face meeting. We mapped institutions offering M&E track/ concentration in MPH programs globally through an online search. The mapping did not involve any primary data collection from human respondents. Google Scholar and PubMed were searched for information including M&E teaching in the form of an independent M&E track/ concentration in MPH, Master of Arts (MA), Post Graduate Certificate and Post Graduate Diploma as programs/courses. Key words for search have been included in Fig. 1.

The search strategy was developed independently by two members of the team and reviewed by an additional third member. The search was conducted by an intern as part of his coursework under the supervision of a team member. This search was supplemented by a snowballing approach to identify institutions offering these programs. We triangulated the results with our results from an earlier activity which identified Masters level programs offering M&E modules or courses. The earlier activity was undertaken in September 2013, to identify all institutions that offered an M&E module in Masters’ level programs globally.

Once a potential university offering M&E track/concentration was identified, detailed information of their M&E curriculum was obtained from university websites and brochures. The data about curricular contents was extracted and compiled in a Microsoft Excel worksheet. The extraction and compilation of the data was done by two team members independently.

In order to determine the frequency of inclusion of a domain within the M&E track/ concentration at the institutes, we used the competency framework adopted by the Association of Schools of Public Health (ASPH) for a Masters degree in public health [7]. Four independent team members reviewed the curriculum of the track/ concentration and matched the course contents to the corresponding core domains^1^ suggested in the ASPH framework. The depth of coverage for a specific core domain was assessed using the Miller’s Triangle as a reference [8]. Miller’s Triangle is divided into four progressive steps of competency ranging from ‘knows’ to ‘does’ (1. *Knows:* This is the knowledge one must have to be able to fulfill future tasks; 2. *Knows how:* This level indicates whether the student knows how to use the knowledge; 3. *Shows how:* The student is able to show that he/she can perform in a simulated environment; 4. *Does:* This is acting independently in the complex situation of an everyday context).

The methodology for the analysis of the curricular content was finalized with an aim to minimize subjectivity and interpretation errors of the curriculum reviewers. Each available curriculum was independently reviewed by four reviewers. We standardized the reviewer’s knowledge in M&E by requesting all of them to complete a standard course on M&E fundamentals available on the MEASURE Evaluation website [9]. These reviewers sat together for a half-day meeting and worked on a dummy curriculum and fitted these into the ASPH core-competency framework. This was followed by a similar exercise in assessing the depth of the core-competencies using the Miller’s triangle. The reviewers were aware of the Miller’s triangle since their academic work had familiarized them with its contents. We had reviewed similar frameworks such as those suggested by UNAIDS for HIV competencies and other agencies, but chose the Miller’s Triangle for its simplicity and ease of usage, as well as its easy understandability among academicians and program coordinators. We modified the scaling of the Miller’s Triangle from its four suggested levels to a linear scale from zero through ten where one represented the extreme end of ‘knows’ and ten represented a handling of the competency at the extreme of the ‘does’. A similar half-day meeting was organized to familiarize the reviewers with the assessment of depth of teaching. After the responses were discussed and standardized, each reviewer was then given a copy of all the M&E track/ concentration curricula and assigned a time-frame of three weeks to complete the preliminary curricular review. Any discrepancy among the reviewers’ scores for either the determination of the frequency of inclusion of a domain or the level of depth of the domain was resolved through discussion within the entire team.

Identification of core competencies for an M&E track/ concentration in the South Asian context was undertaken through a group exercise where the participants from the 11 institutions were divided into four groups. Each group with the help of a facilitator arrived at a list of draft core competencies. The discussions in the group were aided by providing the group with frameworks and reference materials for drafting competencies. The groups also had access to the results of the curricular review undertaken by the core team. The expert group predominantly comprised of academicians with more than 10 years of experience and those who engaged in either leading the M&E teaching within their institutions or occupying a senior academic/ leadership position within the institution. The profiles of these experts are included in Table 1.

**Table 1:** Profiles of experts in Consultation meeting

Sr. No.	Designation	Expert’s age	Gender	Current work / responsibility
1	Lecturer, Department of Public Health	<35 years	Female	Academics and Research
2	Professor, Community Medicine	35-45 years	Male	Associate Dean (International Health)
3	Adjunct Professor, School of Health Systems and Public Health	>45 years	Male	Academics and M&E expert
4	Program Officer	<35 years	Female	Program Administration
5	Faculty, Department of Public Health	35-45 years	Female	Academics and Research
6	Head, Continuing Education Program (CEP) & mHealth	>45 years	Male	Implementation and M&E expert
7	Intern	<35 years	Male	Occupational Therapist
8	Senior Instructor, School of Public Health & Community Medicine	35-45 years	Female	Academics
9	Professor	>45 years	Male	Academics and M&E expert
10	Capacity Building Specialist	>45 years	Female	M&E expert
11	Faculty	35-45 years	Male	Pedagogy, Academics and Research
12	Programme Manager	<35 years	Male	M&E implementation
13	Lecturer II and Internship Coordinator	35-45 years	Male	Academics & Research
14	Professor and Head of Department	>45 years	Female	Academics & Research
15	Assistant Professor	35-45 years	Male	M&E expert
16	Professor and Head	>45 years	Male	Health administration and program management
17	Faculty	<35 years	Female	Epidemiology and Research
18	Professor	35-45 years	Male	Academics and M&E expert
19	Associate Professor	35-45 years	Male	Psychology and Program Implementation
20	Professor and Head of Department	>45 years	Male	Health administration, Academics and Research
21	Professor & Head of Department	>45 years	Male	Academics, Pedagogy and Evaluation
22	Ministry of Health	>45 years	Male	M&E expert and Program Implementation
23	Faculty	35-45 years	Male	M&E expert, Health Information Systems
24	Program Officer	<35 years	Female	Program Administration
25	Head - M&E Unit	<35 years	Female	M&E implementation, Quality Assurance
26	Professor & Head	>45 years	Male	M&E expert and Program Implementation
27	Professor and Director	>45 years	Male	Public Health Education, Academics and Research
28	Professor and Head	>45 years	Male	M&E expert
29	Associate Professor	>45 years	Male	Academics and Research
30	Team Lead	>45 years	Female	M&E and Competency Framework expert

The lists of draft core competencies produced by each group were subjected to a voting exercise, where all experts voted on each competency statement. The experts voted for each competency either as ‘core’ (must be included), ‘additional’ (maybe included) or ‘not to be included’; using different colored stickers. A brainstorming and consensus building exercise was subsequently undertaken wherein core competency statements were revised to consider issues such as duplication, overlap of core competency statements, etc. till the time a consensus was reached by the larger group.

## Results and discussion

Through the curricular review, we identified 21 programs offering an M&E track/ concentration as part of their postgraduate educational program. The list of the institutions is depicted in Table 2 below.

**Table 2:** List of institutions offering M&E track/ concentration as a part of their educational program globally

Sr. No.	Program/ School	University/ Institute	Website	Access date
1	School of Health systems and Public Health	University of Pretoria, South Africa	http://www.up.ac.za/school-of-health-systems-and-public-health	July 28, 2015
2	Continuing education	University of Pretoria, South Africa	http://ce.up.ac.za/default.aspx?tabid=58&Course=1a2e86f3-b8f2-df11-9e88-0050569b0004	July 28, 2015
3	School of Public Health	Kenyatta University	www.ku.ac.ke/schools/public_health/department/department-of-community-health	July 28, 2015
4	School of Health Sciences	Mount Kenya University	http://www.mku.ac.ke/index.php/academics-programmes	July 28, 2015
5	School of Public Health & Tropical Medicine	Tulane University	http://www.sph.tulane.edu/publichealth/academics/index.cfm	July 28, 2015
6	University of Western Cape	University of Western Cape	http://www.uwc.ac.za/Pages/AllProgrammes.aspx	July 28, 2015
7	Department of Sociology and Social Anthropology	Stellenbosch University	http://www.samea.org.za/Training-0.phtml	July 28, 2015
8	University of Forte Hare	University of Forte Hare	http://www.university-directory.eu/js/createpage/0/program-courses/Masters-degrees/all-disciplines/alldisciplines/University+of+Fort+Hare+(UFH)/ZA/5058/Masters+Degrees#courseheader	July 28, 2015
9	Cavendish University	Cavendish University	www.cavendishza.org/index.php?option=com_content&view=article&id=83&Itemid=136	July 28, 2015
10	Uganda Management Institute	Uganda Management Institute	http://www.umi.ac.ug/academic-programmes	July 28, 2015
11	College of Business and Management Sciences	Makerere University	http://bams.mak.ac.ug/	July 28, 2015
12	Dept. of Economics & Development Studies	Mount Kenya University	http://www.mku.ac.ke/index.php/academics-programmes/118-programmes/397-post-graduate-diploma-in-monitoring-and-evaluation-pgd-m-e	July 28, 2015
13	Daystar university	Daystar university	http://www.daystar.ac.ke/index.php	July 28, 2015
14	Dept. of Health Services Management and the International Center for Monitoring and Evaluation	Jimma University	www.ju.edu.et/cphms/node/47?q = node/86	July 28, 2015
15	Department of Economics & Development studies	Mount Kenya University	http://www.mku.ac.ke/index.php/academics-programmes/118-programmes/396-master-of-arts-monitoring-and-evaluation-abbreviated-as-m-a-m-e	July 28, 2015
16	Population Studies and Research Institute	University of Nairobi	http://sphun.uonbi.ac.ke/sites/default/files/chs/commhealth/commhealth/MPH%20CURRICULUM.pdf	July 28, 2015
17	Centre for Health Policy, Programs and Economics, Melbourne School of Population	University of Melbourne	https://handbook.unimelb.edu.au/view/2013/%21244-CW-SPC%2B1003	July 28, 2015
18	Department of Health Studies and Gerontology	University of Waterloo	https://uwaterloo.ca/public-health-and-health-systems/future-graduate-students/professional-programs/master-health-evaluation/curriculum	July 28, 2015
19	Faculty of Health Sciences	University of Cape Town	http://www.publichealth.uct.ac.za/phfm_master-public-health	July 28, 2015
20	Faculty of Public Health	Mahidol University	http://www.ph.mahidol.ac.th/Webpages_MPH/	July 28, 2015
21	InstitutoNacional de SaludPública	INSP, Mexico	www.insp.mx/education/the-school-of-public-health-of-mexico.html	July 28, 2015

**Fig. 2:**
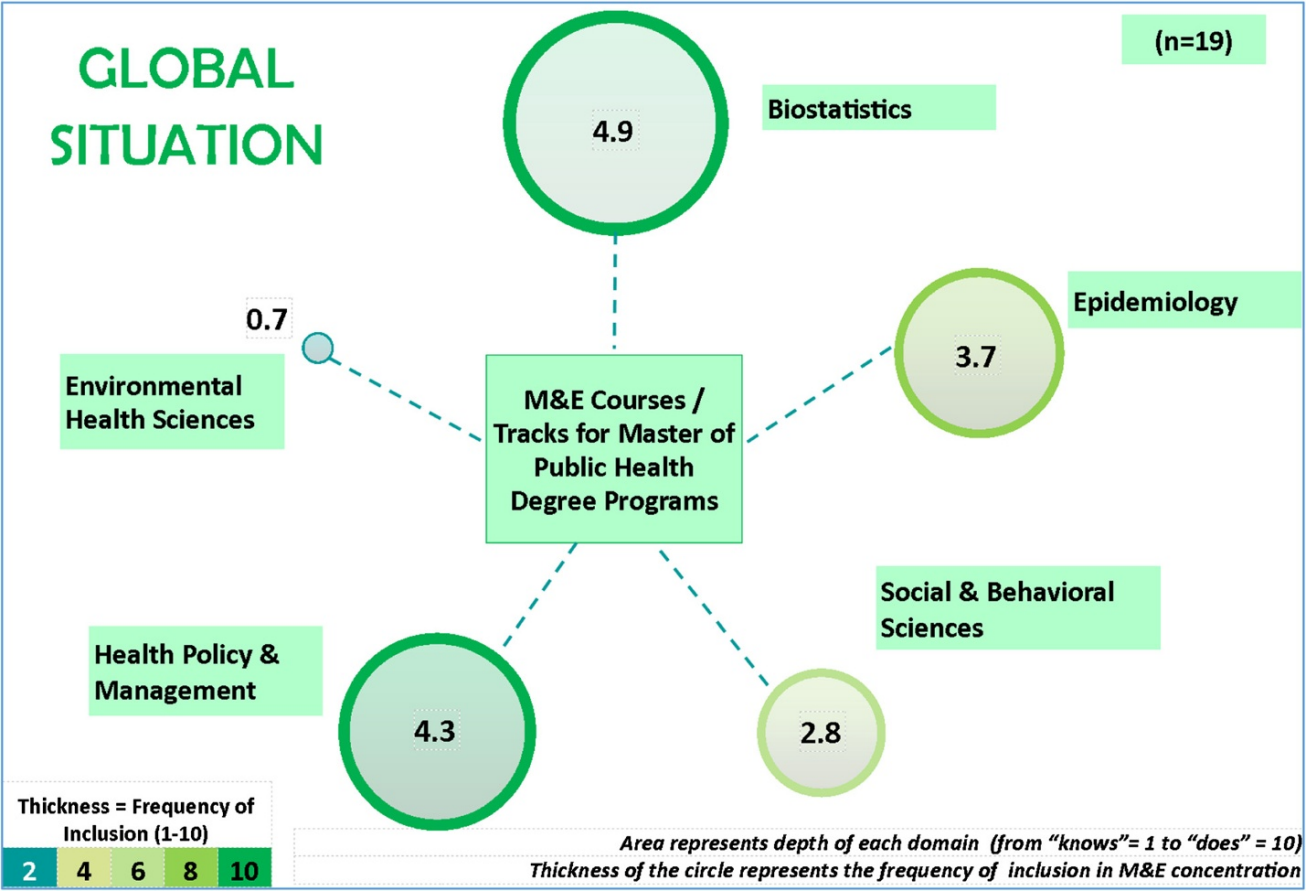
Global situation: Curricular content and depth across M&E tracks/ concentrations

We included 19 programs which offered detailed information permitting a curricular review. The results of the frequency and depth of the domains have been depicted in Fig. 2 below, where the five core ASPH domains are depicted as circles. The thickness of the boundary for each circle represents the frequency of inclusion of each domain within the curricula of the included institutes. It is evident from the figure that majority of the curricula of M&E tracks/ concentrations currently include Biostatistics. This is closely followed by the Health Policy and Management domain. The Environmental Health Sciences domain has the lowest representation in M&E tracks/ concentrations.

The area of each circle represents the depth of inclusion of the curricular content in that domain. As Fig. 2 suggests, both Biostatistics and the Health Policy and Management domains are covered in much greater depth across M&E tracks/ concentrations globally as compared to the other domains. The Environmental Health Sciences domain is covered with the least depth among these five core domains.

Figure 2 represents the depth of each domain that currently exists in M&E tracks/concentrations. Whether this current admixture of the level of domains is an appropriate mix or what is an appropriate mix was not a direct output of the current activity.

The list of core competency statements for M&E tracks/concentrations that were agreed upon by the group is as follows:

1. Ability to develop/use M&E tools with special reference to National Health Programs
2. Ability to develop and design framework and link the indicators with frameworks
3. Ability to identify the sources of data, collect, manage, analyze and interpret data
4. Ability to assess and maintain quality of data
5. Ability to comprehend M&E concepts and importance of M&E & differentiate between M&E
6. Ability to identify, develop and evaluate indicators
7. Ability to identify and engage stakeholders at all levels
8. Ability to identify evaluation designs and conduct an evaluation
9. Ability to write reports, communicate & disseminate M&E information
10. Ability to manage and lead the M&E team
11. Ability to critically appraise the M&E system
12. Ability to identify appropriate principles and guidelines to ensure ethical conduct of M&E
13. Ability to use M & E data to support decision making, advocacy and other purposes
14. Ability to design M&E systems in terms of social (social stratification), economic, and cultural context
15. Ability to develop and implement a M&E Plan

The competency-driven approach towards public health education is suggested as an indispensable component of public health education [10, 11]. It addresses the supply-side of the health systems and works towards creating the right balance of skill-sets among future public health professionals.

According to Le Boterf as reported in Brahimi [12] “the concept of competency has to be consistent with the changing contexts and situations in the workplace.” Since developing countries differ from developed countries in their public health challenges, there are differences in healthcare delivery and the competencies that are expected of these graduates. There are concerns about whether existing programs in low and middle-income countries (LMIC) equip public health alumni to be effective, and whether the taught competencies from these programs are relevant to their contexts [13]. The multidisciplinary learnings of public health bring together people with diverse professional qualifications who have to rely on each other for their day-to-day functioning. These public health professionals should have the ability to understand the problem, while possessing an ability to identify and implement efficient solutions. This multidisciplinary approach towards addressing public health concerns demands complex skills among public health professionals. Competency frameworks are the building blocks that help structure training programs that lead to the acquisition of relevant skills.

These competency frameworks should be designed while remembering that a properly designed MPH degree is expected to be intellectually challenging, with emphasis on active, student-centered learning, problem solving and acquisition of essential public health practice skills [14]. The development of competency frameworks [15–17] is ideally guided by sound research and consultation, is evidence-informed and yet flexible enough to be adopted across diverse institutional settings. Competency frameworks are however scarcely documented for public health programs in developing countries. There have been limited efforts in the South Asian context to develop public health competencies for under-graduate and post-graduate public health education.

The program contents in M&E tracks/ concentrations would be expected to show variability in the reported curricula. This variability could represent an actual difference in the curricular contents between the programs; or a poor reporting of what actually gets covered as a part of the teaching. While the latter continues to remain a limitation of our work, we addressed the issue of accurately reporting the curricular contents against a standard framework. The group chose to proceed with the ASPH core competency model for MPH programs [15] as a standard reference against which we judged the curricular contents included within an M&E track/ concentration. The ASPH core competency model is universally recognized, widely adhered to and has been developed through a rigorous methodology.

The inclusion of a particular topic within the curricular contents of a specific academic program was an area of potential subjective variations in its analysis.

Our curricular review showed that different domains of the framework are covered differently across the programs. The quantitative sciences (Biostatistics and Epidemiology) and Health Policy and Management are covered in much greater depth than the other two domains (Social & Behavioral Sciences and Environmental Health Sciences). This is attributable to the greater ‘hands-on doing’ exercises and working on real-time datasets that gets reflected in the quantitative sciences, particularly Biostatistics. This has resulted in the quantitative sciences getting a higher score in the depth of coverage along with health systems. The M&E track/ concentration had a higher frequency of inclusion of these three domains when compared to the Social & Behavioral Sciences and Environmental Health Sciences as is evident by the thickness of the circle boundaries.

The structure and duration of an ideal M&E track/ concentration for a country or a region could vary considerably depending on the needs and structure of the health system, the health system priorities and the ability of health professional educational systems to respond to these priorities. Although our work drew upon the experiences of several senior experts, there is scope for further fine-tuning once there is some experience in delivering these competencies through an academic program.

The limitations of our work include the presence of subjectivity in assessing the curriculum. We partly addressed it through independent review of the curriculum by multiple team members. Inconsistencies or differences in results were discussed and addressed. We looked only at the core ASPH MPH competency domains, with a consequent difficulty in classification of curricular contents completely within one specific domain. This work is based on *what is documented* while in an ideal scenario, we would have wanted to witness *what is carried out or delivered* as a part of the academic experience. An M&E track/ concentration is housed within an MPH program and it is difficult to judge the merit of a track through examining a track alone. Several deficiencies within a track could have been addressed through other core or elective teachings across the program.

## Conclusions

This work presents an opportunity for institutions to identify and re-examine their M&E competencies as a part of their specialized tracks within MPH programs. Our curricular analysis approach has the potential for adaptation and further use in curriculum analysis across different academic specialties.

## Footnotes

1 This ASPH model includes five core discipline-specific domains: Biostatistics, Environmental Health Sciences, Epidemiology, Health Policy Management, and Social and Behavioral Sciences.
